# Antibacterial Prodrugs to Overcome Bacterial Antimicrobial Resistance

**DOI:** 10.3390/ph17060718

**Published:** 2024-06-01

**Authors:** Catarina Maria, Ana M. de Matos, Amélia P. Rauter

**Affiliations:** Centro de Química Estrutural, Institute of Molecular Sciences, Faculdade de Ciências, Universidade de Lisboa, 1749-016 Lisboa, Portugal; cbmaria@fc.ul.pt (C.M.); amamatos@fc.ul.pt (A.M.d.M.)

**Keywords:** antimicrobial resistance, prodrug development, antibacterial prodrugs, tuberculosis, gram-negative bacteria, gram-positive bacteria

## Abstract

Antimicrobial resistance (AMR) is an increasingly concerning phenomenon that requires urgent attention because it poses a threat to human and animal health. Bacteria undergo continuous evolution, acquiring novel resistance mechanisms in addition to their intrinsic ones. Multidrug-resistant and extensively drug-resistant bacterial strains are rapidly emerging, and it is expected that bacterial AMR will claim the lives of 10 million people annually by 2050. Consequently, the urgent need for the development of new therapeutic agents with new modes of action is evident. The antibacterial prodrug approach, a strategy that includes drug repurposing and derivatization, integration of nanotechnology, and exploration of natural products, is highlighted in this review. Thus, this publication aims at compiling the most pertinent research in the field, spanning from 2021 to 2023, offering the reader a comprehensive insight into the AMR phenomenon and new strategies to overcome it.

## 1. Introduction

Antimicrobial resistance (AMR) has been increasing over recent decades, posing a significant threat to both human and animal health. AMR arises from the evolution of bacteria, viruses, fungi, and parasites, which cease to respond to antimicrobial agents. The misuse and excessive intake of antibiotics, along with the lack of information about AMR, contribute to the development of this threat [1,2,3].

### 1.1. Bacteria and Bacterial AMR

Bacteria are the most ancient and abundant forms of life on Earth, being, alongside archaea, the only organisms characterized by a prokaryotic cellular organization. These organisms are responsible for several fundamental functions of ecosystems, making life on Earth possible [4].

In terms of cell envelope constitution, bacteria are divided into two groups: Gram-negative bacteria (GNB) and Gram-positive bacteria (GPB). GNB envelope is composed of three layers: a protective outer membrane (OM), a thin peptidoglycan (PG) cell wall, and an inner membrane (IM), while GPB consist only of a thick protective PG cell wall surrounding the plasma membrane. The OM of GNB exhibits lipopolysaccharides (LPS) bound to its outer leaflet, and phospholipids bound to its inner leaflet. Additionally, this unique GNB feature presents proteins that allow small molecules to cross the membrane. In both GNB and GPB, the PG cell wall is presented as a rigid exoskeleton responsible for bacterial cell shape, being composed of repeat units of *N*-acetylglucosamine–*N*-acetylmuramic acid (GlcNAc-MurNAc). Finally, the inner plasma membrane consists of a phospholipid bilayer responsible for bacterial structure, transport, and biosynthetic functions [5,6].

The World Health Organization (WHO) recognized the issue of bacterial AMR as one of the top ten health threats and reported a list of priority pathogens in 2022, including *Mycobacterium tuberculosis* (*M. tuberculosis*) strains. Also, three other priority groups were identified. The critical group covers carbapenem-resistant *Acinetobacter baumannii* (*A. baumannii*), carbapenem-resistant *Pseudomonas aeruginosa* (*P. aeruginosa*), and carbapenem- and 3rd generation cephalosporin-resistant *Enterobacteriaceae*. The high priority group includes vancomycin-resistant *Enterococcus faecium* (*E. faecium*), clarithromycin-resistant *Helicobacter pylori* (*H. pylori*), fluoroquinolone-resistant *Salmonella* species, vancomycin- and methicillin-resistant *Staphylococcus aureus* (*S. aureus*), fluoroquinolone-resistant *Campylobacter* species, and 3rd generation cephalosporin- and fluoroquinolone-resistant *Neisseria gonorrhoeae* (*N. gonorrhoeae*). Finally, the medium priority group is composed of penicillin-non-susceptible *Streptococcus pneumoniae* (*S. pneumoniae*), ampicillin-resistant *Haemophilus influenzae* (*H. influenzae*), and fluoroquinolone-resistant *Shigella* species. The emergence of multidrug-resistant (MDR) bacterial strains, namely *E. faecium*, *S. aureus*, *Klebsiella pneumoniae* (*K. pneumoniae*), *A. baumannii*, *P. aeruginosa* and *Enterobacter* species (ESKAPE), and extensively drug-resistant (XDR) pathogens resulted in drug inefficacy [7,8].

Recently, an increase in bacterial AMR was reported and attributed to preventive treatment of hospitalized COVID-19 patients to decrease the risk of contracting bacterial infections. Also, it is expected that this concerning phenomenon will lead to the death of 10 million people per year and have a global cost of USD 100 trillion on lost output by the year 2050 [2,3,9,10].

Thus, new antibacterial treatments able to cope with resistance mechanisms are urgently required.

### 1.2. Antibacterial Prodrugs

New antibacterial agents should meet at least one of the innovation criteria defined by the WHO, namely, to act on a new target, to exhibit new modes of action, to present no evidence of cross resistance, or to belong to a new class of antibiotics [7]. Also, understanding bacterial resistance mechanisms, such as efflux pump overexpression, porin channel loss, mutations, antibiotic uptake reduction, and antibiotic alteration or degradation, is essential to developing new antibacterial agents [5,6].

Prodrug development is emerging as a popular strategy to address the challenges encountered in drug development. Fifty prodrugs have been approved by the US Food and Drug Administration (FDA) between 2012 and 2022, indicating their growing acceptance [11,12]. This approach aims at mitigating issues such as the sub-optimal pharmacokinetic, biophysical, and physiochemical properties commonly found in drug candidates, including poor solubility, poor membrane permeability, chemical instability, and non-selective delivery, limiting their efficacy.

A prodrug is a compound that initially lacks pharmacological activity, becoming active through conversion into an active structure and non-toxic species upon intake. This type of drug can be classified into three classes: (i) carrier-linked prodrugs; (ii) bioprecursor prodrugs; and (iii) double prodrugs [11,12,13]. Modifying fundamental properties like polarizability, hydrophilicity, electronic and steric factors, hydrogen bonding, and chemical reactivity can yield the desired drug behavior [11,12].

Despite its promise, developing an ideal prodrug is challenging as it must meet several criteria without compromising final compound efficacy, including adequate aqueous solubility, optimal conversion rate, good safety profile, safe promoiety without undesirable side effects, rapid excretion, membrane permeability, and adequate stability. Moreover, although in vitro and in vivo assays have been developed over the years to evaluate the clinical potential of prodrugs, the development of these compounds is usually more complex and less predictable than for other drugs [11,12].

In the field of antibacterial agent development, the prodrug approach is also emerging as a popular strategy [13].

Hence, in addition to the carbohydrate-based prodrugs recently reviewed [14], we now aim at presenting the most recent findings on all chemical entities known to act as antibacterial prodrug agents reported from 2021 to 2023.

## 2. Advancements in Antibacterial Prodrug Applications

### 2.1. Antituberculosis Prodrugs

The cell wall of *M. tuberculosis* is a distinctive feature of mycobacteria. Despite being classified as GPB, these bacteria exhibit a structure reminiscent of GNB, featuring an OM with mycolic acids [15].

Treatment of tuberculosis (TB) is carried out based on combination therapy of first-line drugs, i.e., isoniazid, rifampicin, ethambutol, and pyrazinamide. MDR-TB, characterized by isoniazid and rifampicin resistance, is widespread, while XDR-TB, characterized by resistance to second-line drugs, namely quinolones and aminoglycosides, is increasing [16,17,18]. The emergence and spread of these drug-resistant strains highlights the urgency of finding new antibacterial agents and new modes of action.

**Figure 1:**
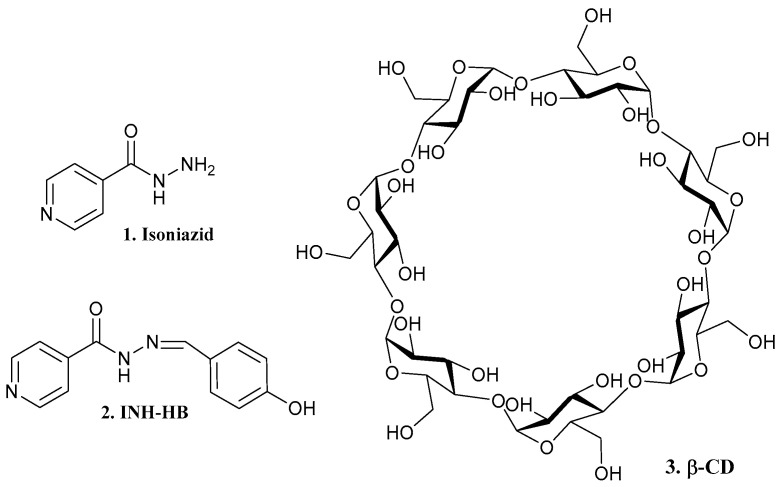
Structure of isoniazid (**1**), INH-HB (**2**), and β-CD (**3**) [16,17,18].

Isoniazid (**1**, Figure 1), a prodrug activated by the action of the enzyme KatG, inhibits InhA protein, disturbing *M. tuberculosis* cell wall biosynthesis. Resistance to isoniazid may be achieved through several mechanisms; however, it is mostly attributed to a mutation in KatG or in the InhA-binding site [16,17]. In the last two years, efforts have been made to understand and counter isoniazid resistance mechanisms. In 2021, the design and synthesis of new ‘direct’ InhA inhibitors that did not require KatG activation were reported [16] and, also, a new potential isoniazid inactivation mechanism was disclosed [14,17]. Since acetylation is a well-known mechanism used by bacteria to modify drugs and drug targets, Arun et al. [17] hypothesized that *M. tuberculosis* could adopt a similar mechanism to inactivate isoniazid. Purified Rv2170, a putative *M. tuberculosis* acetyltransferase, was then used to test this hypothesis and it was found that it was able to acetylate isoniazid in vitro, which was then hydrolyzed to isonicotinic acid and acetyl hydrazine. Furthermore, the treatment of isoniazid with the enzyme resulted in a failure to kill *M. smegmatis* bacteria. This work suggested that the acetylation of the drug mediated by Rv2170 could be a novel strategy adopted by isoniazid-resistant *M. tuberculosis* strains, which is an interesting finding for future antituberculosis drug development.

To cope with antibacterial resistance mechanisms, the formulation of lipid-based nanoparticles encapsulating drugs and prodrugs has become a promising approach for allowing targeted drug delivery. Also, the formulation of cyclodextrin drug complexes has been studied for improvement of lipid encapsulation of hydrophobic drugs. Safari et al. [14,18] reported the synthesis of INH-HB (**2**, Figure 1), a hydrophobic isoniazid (INH) prodrug resulting from its coupling with 4-hydroxybenzaldehyde (HB). Moreover, prodrugs’ complexation with α, β, and γ-cyclodextrins was tested and β-cyclodextrin (β-CD, **3**, Figure 1), with a predicted formation of H-bonding interactions between its hydroxy groups and the pyridinic nitrogen atom of the prodrug, was found to be the most suitable for increasing INH-HB solubility in water; therefore, being then chosen for further liposome encapsulation. The complex INH-HB@CD-in liposomes promoted higher INH release rates in acidic media when compared to neutral pH media. Thus, after liposome phagocytosis by macrophages, where *M. tuberculosis* is usually found, this new anti-TB system was revealed as a promising formulation to target the intracellular delivery of INH.

**Figure 2:**
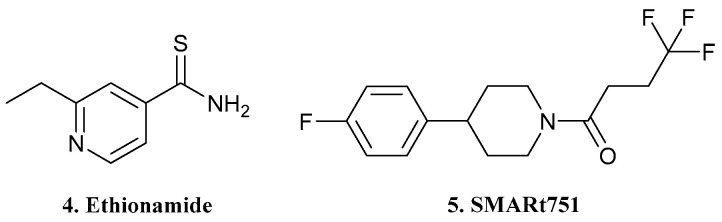
Structure of ethionamide (**4**) and SMARt751 (**5**) [19].

Ethionamide (**4**, Figure 2) is also an antituberculosis prodrug, and is activated by the monooxygenase EthA, which inhibits InhA. Mutations in the *ethA* gene are responsible for bacterial resistance to ethionamide. SMARt751 (**5**, Figure 2), a small molecule reported by Baulard’s group [19] in 2022, stimulates the *mymA* operon that encodes a monooxygenase activating ethionamide. This molecule was shown to potentiate the efficacy of the prodrug in both in vitro and in vivo acute and chronic TB mouse models. Moreover, the reversal of ethionamide resistance by SMARt751 was determined by using a set of thirty-seven *M. tuberculosis* clinical isolates with a minimum inhibitory concentration (MIC) of ethionamide of ≥4 µg/mL. In thirty-three of these isolates, treatment with 150 nM SMARt751 reduced the MIC value to ≤0.8 µg/mL. Among these, fourteen isolates required only 10 nM to achieve the same reduction. Additionally, a highly resistant isolate, with an ethionamide MIC value of ≥256 µg/mL, showed a MIC reduction to 0.8 µg/mL after treatment with 300 nM SMARt751. Finally, through a model that extrapolates animal pharmacokinetic and pharmacodynamic parameters to humans, the group predicted that a daily dose of 25 mg of SMARt751 could allow a four-fold reduction in the administered dose of ethionamide, maintaining the same efficacy and reducing side effects. Thus, the authors concluded that ethionamide-SMARt751 combination therapy could not only be used as a second-line treatment for TB but also replace isoniazid and become a new first-line drug for the treatment of the disease [19].

**Figure 3:**
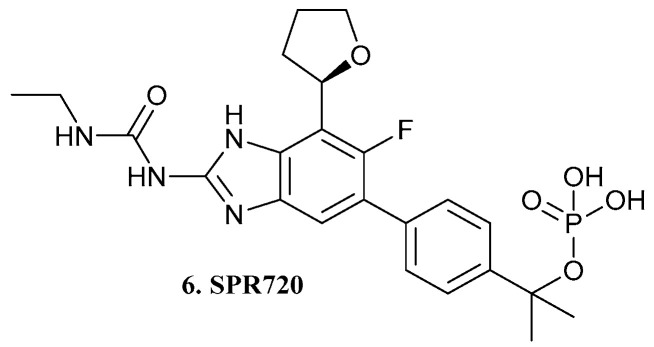
Structure of SPR720 (**6**) [20].

Currently, the DNA gyrase inhibitor SPR720 (**6**, Figure 3), a stable phosphate prodrug of SPR719, is under development for the treatment of pulmonary tuberculosis and nontuberculous mycobacterial pulmonary disease. In vivo, this new aminobenzimidazole prodrug converts into SPR719, targeting the ATPase subunits of bacterial DNA gyrase. A phase I clinical trial (NCT03796910) was conducted to evaluate the safety, tolerability, and pharmacokinetics of SPR720/SPR719, predicting that a once-daily administration of SPR720 could promote SPR719 therapeutic exposure [20].

**Figure 4:**
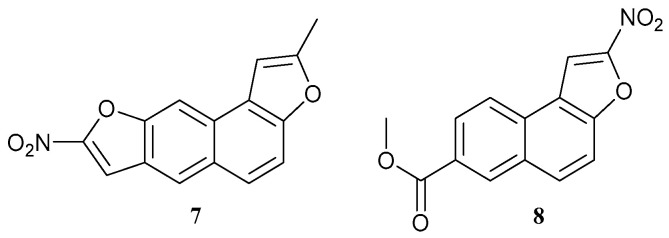
Structure of nNF prodrugs **7** and **8** [21].

In 2023, Cioetto-Mazzabò et al. [21] reported the SigH/Mrx2 stress-response pathway as a promising target for further antituberculosis drug development. Fourteen nitronaphthofurans (nNFs) with mycobactericidal activity were identified and, amongst them, **7** and **8** (Figure 4) exhibited the highest activity. These compounds presented MIC_90_ values of 0.062 and 0.125 µg/mL against *M. tuberculosis*, respectively. Both compounds were considered safe, with 50% cytotoxic concentration (CC50) values of 75.77 and 64.9 µg/mL determined, respectively. Moreover, the selectivity indices (CC50/MIC_90_) for compounds **7** and **8** were determined, exhibiting values of 1211.5 and 519.2, respectively.

nNFs are prodrugs that require activation mediated by the coordinated action of *sigH* and *mrx2* genes within tuberculosis bacilli. The action of these prodrugs induces the SigH stress response which, in turn, induces *mrx2* overexpression. Mrx2 then activates these agents, allowing the eradication of both replicating and non-replicating *M. tuberculosis* strains [21].

### 2.2. Prodrugs against Gram-Negative Bacteria

**Figure 5:**
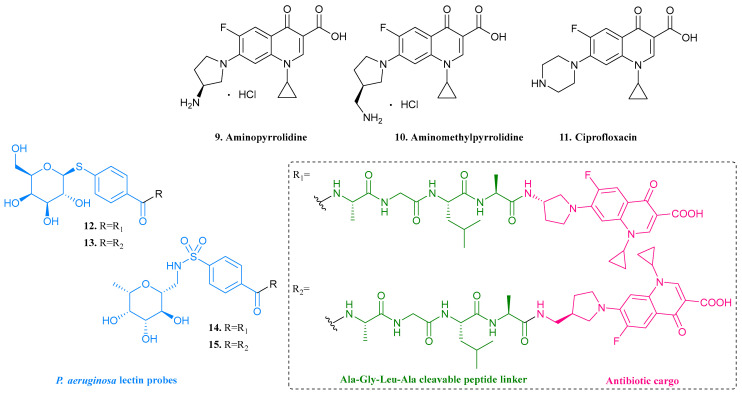
Structure of aminopyrrolidine (**9**), aminomethylpyrrolidine (**10**) ciprofloxacin (**11**), and biofilm-targeted prodrugs **12**–**15**. *P. aeruginosa* lectin probes, cleavable peptide linkers and antibiotic cargos are highlighted in blue, green and pink, respectively [22].

Fluoroquinolone antibiotics are often used in the treatment of GNB infections. However, fluoroquinolone-resistant pathogens are emerging and are included in the WHO priority list [9,22,23]. *P. aeruginosa* strains exhibit several resistance mechanisms to a broad spectrum of antibiotics, leading to life-threatening infections. The formation of biofilms exhibiting bacterial lectins LecA and LecB as key structural components, contributes the most to the pathogenicity of these bacteria. Thus, Meiers et al. [14,22] reported the synthesis and mechanism of action of the first biofilm-targeted prodrugs obtained by linking *P. aeruginosa* lectin probes to antibiotic cargos through alanine-glycine-leucine-alanine (Ala-Gly-Leu-Ala) cleavable peptide linkers, as depicted in Figure 5. Fluoroquinolone-based antibiotic cargos, namely aminopyrrolidine (**9**, MIC = 0.027–0.054 µM, Figure 5) and aminomethylpyrrolidine (**10**, MIC = 0.29–1.45 µM, Figure 5), are similar to the potent antibiotic ciprofloxacin (**11**, MIC = 0.125–0.25 µM, Figure 5) in terms of structure and antibiotic profiles; however, these contain a primary amine that may play a crucial role in proteolysis. The developed prodrugs led to drug release and accumulation at the infection site, reducing the side effects attributed to fluoroquinolones while weakening the biofilm structure and contributing to breaking *P. aeruginosa* resistance mechanisms. Within synthesized compounds, thiogalactoside prodrugs **12** and **13** (Figure 5), and *C*-glycoside prodrugs **14** and **15** (Figure 5), were identified for their efficient antibiotic cargo release in 3 h. Also, compounds **12** and **14** have shown a remarkable antibacterial activity, exhibiting MIC values ranging from 0.098 to 0.195 µM. Their efficacy nearly matched that of the parent fluoroquinolone antibiotic cargo **9**.

**Figure 6:**
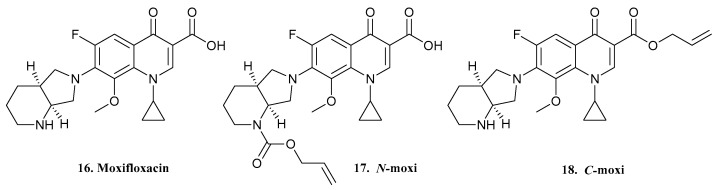
Structure of moxifloxacin (**16**), *N*-moxi (**17**), and *C*-moxi (**18**) [23].

Southwell et al. [23] also reported the use of fluoroquinolone derivatives. Bacteria acquire iron through the secretion of siderophores and, unlike bacterial cells, eukaryotic cells do not possess siderophore transporters. Thus, a siderophore-directed mechanism of prodrug activation could be selective towards bacterial cells. In this context, the group proposed the development of siderophore–ruthenium catalyst conjugates to activate moxifloxacin (**16**, MIC= 300 nM, Figure 6) prodrugs, namely *N*-moxi and *C*-moxi (**17** and **18**, Figure 6), both exhibiting MIC values of approximately 100 µM. However, due to solubility issues, *N*-moxi failed in bacterial uptake assays, and, as such, only *C*-moxi was tested in the following studies.

**Figure 7:**
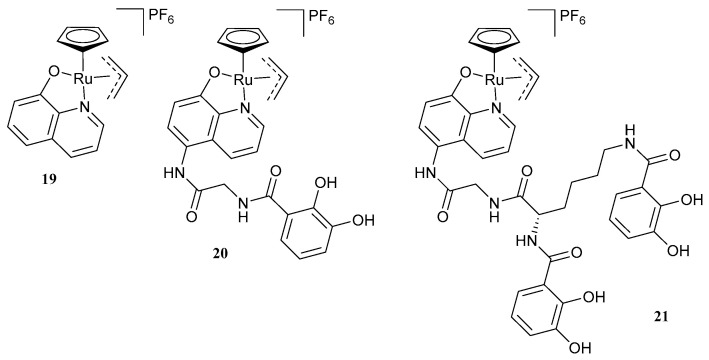
Structure of ruthenium-catalyst **19** and siderophore-linked catalysts **20** and **21** [23].

Aiming at facilitating the internalization of the prodrug-activating ruthenium catalyst (**19**, Figure 7) into bacteria, it was linked to several siderophores presenting different denticities and chelating patterns. Bacterial growth inhibition with incubation of *C*-moxi was investigated. In this study, performed on *E. coli*, all synthesized catalyst–siderophore conjugates exhibited some bacterial internalization. Also, against mammalian cells, ruthenium-containing catalysts were proven nontoxic. Amongst synthesized compounds, siderophore-linked catalyst **20** (Figure 7) promoted a bacterial growth reduction of, approximately, 40%, becoming the most promising catalyst for *C*-moxi activation within a bacterial environment. Compound **21** (Figure 7) was also highlighted. When internalized, it promoted bacterial growth that was completely reversed following the addition of the prodrug, proving its potential to convert *C*-moxi into active moxifloxacin within bacteria. However, the co-addition of the synthesized catalysts and the prodrugs was revealed to be not as efficient as the addition of moxifloxacin, which emphasized that further research is required before considering this approach as a suitable strategy for antimicrobial drug development, e. g., on the compatibility of conjugates with the targeted membrane receptors for internalization and on the stability of the catalyst under aerobic conditions [23].

**Figure 8:**
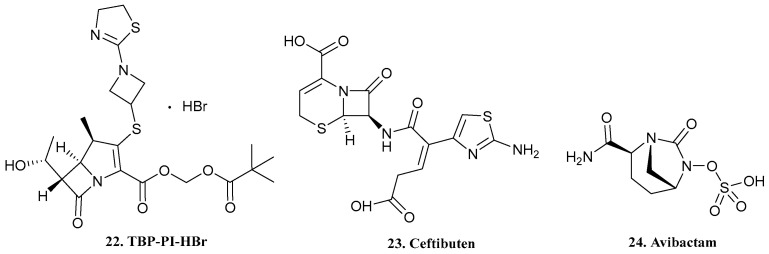
Structure of TBP-PI-HBR (**22**), ceftibuten (**23**), and avibactam (**24**) [24,25,26,27,28,29,30].

Carbapenem-resistant bacterial strains are also included on the WHO priority list [9]. The treatment of urinary tract infections (UTIs) has become a challenge due to the growing presence of MDR bacterial infections, mostly caused by the extended-spectrum *β*-lactamase-producing and quinolone-resistant Enterobacterales [24,25,26,27,28]. Tebipenem pivoxil hydrobromide (TBP-PI-HBr, **22**, Figure 8) is a novel oral tebipenem (TBP) prodrug belonging to the carbapenem class of *β*-lactam antibiotics, which has already shown potent antibacterial activity against different bacterial strains, namely *S. aureus*, *K. pneumoniae*, *S. pneumoniae*, *H. influenzae*, *P. aeruginosa* and *E. coli*, both in vitro and in vivo. TBP-PI was approved in 2009 in Japan for the treatment of ear, nose, throat, and respiratory organs in pediatric patients, with its HBr salt currently under development for the treatment of complicated UTIs due to its activity against Enterobacterales [24,25,26]. Moreover, the repurposing of this prodrug has also been studied for the treatment of infections caused by XDR *Shigella* spp. bacterial strains, TBP exhibiting MIC values ranging from 0.04 to 0.3 µM against different *Shigella* isolates [28].

Ceftibuten-avibactam is a novel, orally available antibacterial combination. Ceftibuten (**23**, Figure 8), an orally active 3rd generation cephalosporin, is already approved for the treatment of infections caused by *H. influenzae*, *H. parainfluenzae*, *M. catarrhalis*, and penicillin-susceptible *S. pneumoniae*, having already shown clinical efficiency in the treatment of uncomplicated UTIs. The addition of the *β*-lactamase inhibitor avibactam (**24**, Figure 8) leads to the broadening of the spectrum of Gram-negative pathogens that are susceptible to the drug, including *β*-lactamase-producing Enterobacterales [27,29,30]. A study carried out by Sader et al. [30] determined MIC_50_ and MIC_90_ values of 0.03 and 0.06 µg/mL, respectively, for the ceftibuten-avibactam combination against 3216 Enterobacterales.

**Figure 9:**
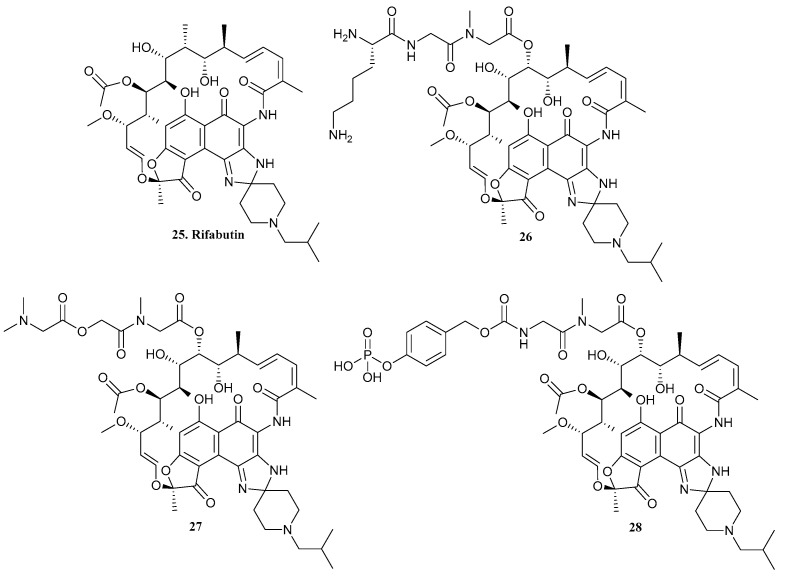
Structure of rifabutin (**25**) and rifabutin-based prodrugs **26**–**28** [31].

Rifabutin (**25**, Figure 9) is a semisynthetic product from the rifamycins class of antibiotics that, in addition to its antituberculosis properties, has recently shown potent activity against *A. baumannii* strains in vitro and in vivo. Due to its limited oral bioavailability, Antraygues et al. [31] reported the development of seventeen water-soluble rifabutin prodrugs for intravenous administration in higher doses. After water solubility, in vitro stability in human plasma, and pharmacokinetic studies, tripeptide prodrug **26**, ester prodrug **27**, and phosphate prodrug **28** (Figure 9) were highlighted for being the most effective in the release of rifabutin, with **26** being particularly emphasized for the highest release over 6 h. Also, the three prodrugs exhibited potent activity (MIC = 0.001 µg/mL) against *A. baumannii*, comparable to rifabutin (MIC = 0.00025 µg/mL), in cation-adjusted Muller Hinton broth (CAMHB) supplemented with human serum, suggesting a serum/plasma mediated release of the active antibiotic. In terms of water solubility, **27** was the only one not exhibiting solubility improvement when compared to the parent drug and, in the context of pharmacokinetic assays, a complete and rapid conversion of **28** into rifabutin was found in mouse plasma samples following intravenous injection. Overall, **26** and **28** seemed to be the most suitable rifabutin prodrugs and are becoming a promising approach to increasing the therapeutic applicability of this antibacterial agent.

**Figure 10:**
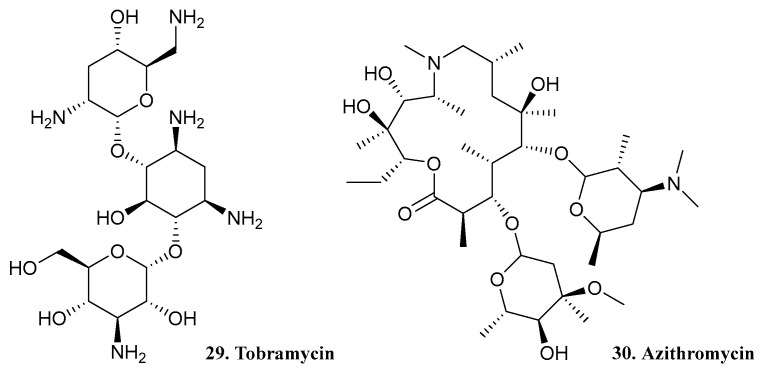
Structure of tobramycin (**29**) and azithromycin (**30**) [32].

**Scheme 1:**
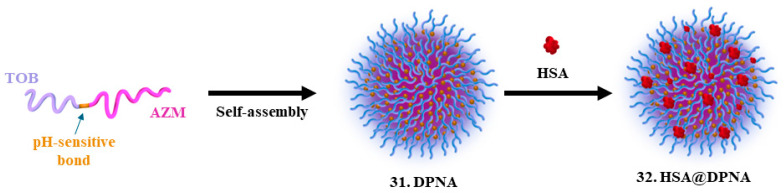
Illustration of the self-assembly of DPNA (**31**) and of the formation HSA@DPNA (**32**) (inspired by the original illustration in [32]).

Nanotechnology has shown promising potential against AMR. Tobramycin (TOB, **29**, Figure 10), an aminoglycoside antibiotic, and azithromycin (AZM, **30**, Figure 10), belonging to the macrolide family of antibiotics, have demonstrated therapeutic effects in cystic fibrosis patients, which are particularly vulnerable to infections caused by *P. aeruginosa* biofilms. In this context, a human serum albumin (HSA)-coated dimeric prodrug-based nanoassembly (DPNA, **31**, Scheme 1) to deliver TOB and AZM was developed by Li et al. [14,32], in 2023. This nanocomplex, named HSA@DPNA (**32**, Scheme 1), was obtained by insertion of pH-sensitive citraconic amide bonds between the two drugs, and exhibited stability under physiological conditions (Scheme 1). For DPNA and HSA@DPNA, pH-dependent antibacterial activity was observed. At pH 7.4, both exhibited MIC values greater than 128 µg/mL. However, at pH 5.5, the MIC values decreased to 1.0 µg/mL, reaching the value determined for the TOB/AZM mixture and suggesting the activation of the prodrug through depolymerization when in contact with an acidic microenvironment. Finally, the quick release of both antibiotics was achieved and therapeutic effects against biofilms in a *P. aeruginosa* infected mouse model, including AZM-promoting biofilm elimination and TOB-eradicating bacteria, were presented, suggesting that this is a candidate for further treatment of biofilm-related infections in vivo.

**Figure 11:**
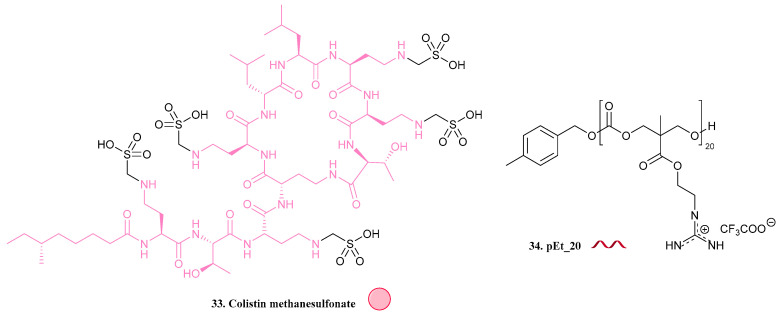
Structure of colistin methanesulfonate (**33**) and pEt_20 (**34**). Colistin moiety is highlighted in pink [34].

**Scheme 2:**
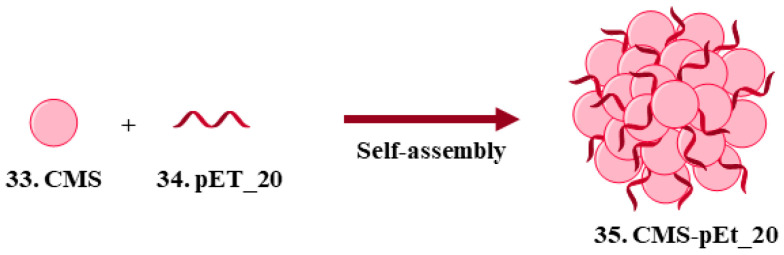
Illustration of the self-assembly of CMS-pEt_20 (**35**) (inspired by the original illustration in [34]).

In the same year, a colistin methanesulfonate (CMS, **33**, Figure 11) nanoassembly was reported; CMS being a colistin prodrug formulated for aerosol administration. Colistin (polymyxin E) is a last-resort drug for the treatment of MDR and XDR Gram-negative infections when other clinically available drugs show no efficacy. However, colistin resistance is emerging and the treatment outcome of resistant strains is compromised [33,34]. In view of overcoming colistin resistance, Zhao et al. [34] developed an anionic CMS and guanidium-functionalized cationic antimicrobial polymer pEt_20 (**34**, Figure 11) nanocomplex, named CMS-pEt_20 (**35**, Scheme 2), expecting the co-delivery of both agents. In vitro assays were performed in *E. coli* CMS-resistant strains with a MIC value of 7.8 µg/mL and, at this concentration, the nanoparticles exhibited biocompatibility. This complex also enabled the eradication of clinically isolated colistin-resistant bacteria, namely, MDR *E. coli* and *K. pneumoniae*, and the reversal of colistin resistance, while the single administration of the polymer or the antibiotic led to no results. Hence, this nanocomplex approach may become a potent strategy to treat colistin-resistant MDR bacterial strains.

**Figure 12:**
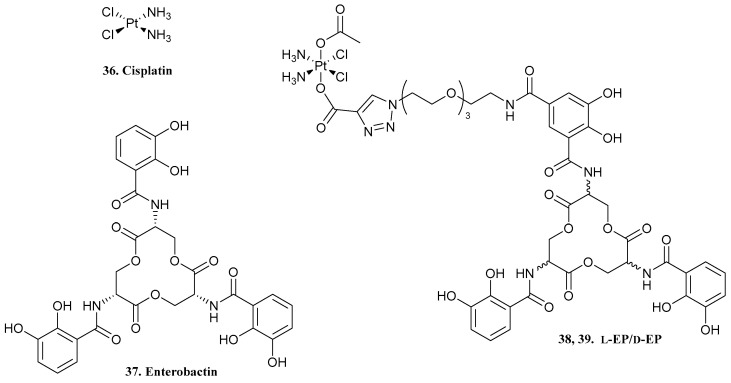
Structure of cisplatin (**36**), enterobactin (**37**), and the prodrugs l-EP (**38**) and d-EP (**39**) [35].

Repurposing of existing drugs is also becoming a promising strategy against AMR, because it is cost-effective and allows the minimization of the risks intrinsic in the process of the development of a new drug [35,36]. In 2022, Guo and Nolan [35] reported the repurposing of the anticancer agent cisplatin (**36**, Figure 12) into an antibiotic by conjugating it to enterobactin (**37**, Figure 12), a siderophore of *Enterobacteriaceae*. The l-EP (**38**, Figure 12) conjugate was obtained, exhibiting antibacterial effects against different *E. coli* strains. This preliminary study demonstrated that l-EP delivery was mediated by enterobactin, while the therapeutic effects were obtained by the reduction of the Pt(IV) prodrug, with the release of cisplatin-active moiety and the consequent growth inhibition and filamentation of *E. coli*. Moreover, substituting l-EP with its enantiomer d-EP (**39**, Figure 12 enhanced antibacterial activity, and treatment with these agents was shown to accumulate ≥10-fold more platin when compared to treatment with free cisplatin. Thus, the potential of the conjugation of a siderophore to cisplatin in antibacterial drug development was demonstrated.

**Figure 13:**
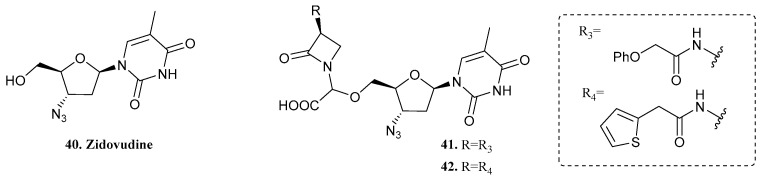
Structure of zidovudine (**40**) and zidovudine-based prodrugs **41** and **42** [36].

**Scheme 3:**
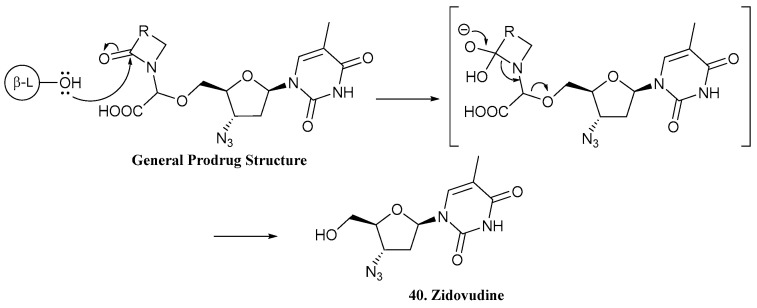
General mechanism of activation of zidovudine prodrugs by β-lactamase enzymes, releasing zidovudine (**40**) [36].

Also, the repurposing of zidovudine (AZT, **40**), an antiviral nucleoside analogue with activity against *E. coli*, was reported by Rosales-Hurtado et al. [14,36] in 2023. In a pioneering study, the group designed and synthesized a small library of ten compounds by covalent linkage of a monobactam promoiety to AZT, which is cleaved by bacterial *β*-lactamase, an intrinsic mechanism of resistance, to release the active AZT, as depicted in Scheme 3. This selective delivery prodrug system has reduced off-target effects and increased therapeutic benefits. Amongst synthesized compounds, **41** and **42** (Figure 13), the most stable ones, were emphasized for their favorable profile for clinical development. The antibacterial activity of both compounds was tested against a panel of isogenic *E. coli* BL21 (DE3)-derived laboratory strains and other clinical isolates producing KPC-3, NDM-1, IMP-1, and *E. cloacae* AmpC *β*-lactamases, using AZT as the reference compound. Isogenic strains were highly susceptible to AZT, exhibiting MIC values ranging from 0.03 to 0.5 µg/mL. The synthesized compounds did not produce significant antibacterial effects against the parent strain *E. coli* BL21 (DE3), with MIC values ranging from 16 to 32 µg/mL. However, isolates with the KPC-3 carbapenemase vector showed the highest susceptibility to both compounds, exhibiting MIC values comparable to that of AZT (MIC for compound **41** = 1 µg/mL; MIC for compound **42** = 0.025 µg/mL), confirming the hydrolysis of the proposed prodrugs by the KPC-type *β*-lactamases.

### 2.3. Prodrugs against Gram-Positive Bacteria

**Figure 14:**
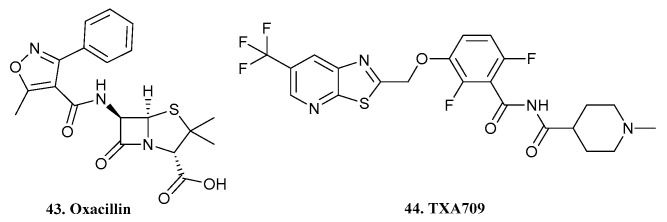
Structure of oxacillin (**43**) and TXA709 (**44**) [37].

Oxacillin (**43**, Figure 14), a member of the *β*-lactam family of antibiotics, was developed for the treatment of staphylococcal infections, namely the ones caused by methicillin-sensitive *S. aureus*. However, it has become ineffective against methicillin-resistant *S. aureus* (MRSA) strains. In this context, Kaul et al. [37] reported a study of the co-administration of oxacillin and the FtsZ-targeting prodrug TXA709 (**44**, Figure 14), with forty-five *S. aureus* isolates intrinsically resistant to oxacillin, including MRSA strains, with MIC values ranging from 128 to 512 µg/mL. The addition of TXA707 (0.5x MIC of TXA707) led to a significant decrease in MIC values for oxacillin, now ranging from 0.063 to 0.5 µg/mL against all isolates. TXA707 and oxacillin demonstrated no antibacterial activity when tested alone, suggesting a potentiation of the oxacillin effect by the Ftz-targeting prodrug.

**Figure 15:**
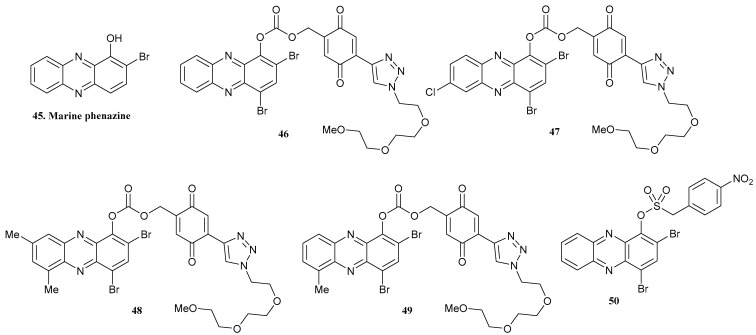
Structure of marine phenazine (**45**), carbonate-linked HP prodrugs **46**–**49**, and nitroarene-based HP prodrug **50** [38,39].

In 2023, Huigens III’s group reported the synthesis of halogenated phenazine (HP) antibacterial prodrugs, inspired by the structure of 2-bromo-1-hydroxyphenazine (marine phenazine, **45**, Figure 15), a natural product with potent activity against *S. aureus* and *S. epidermis* bacterial strains (MIC = 6.25 µM). The group discovered that the addition of another bromine atom at position-4 of the phenazine heterocycle could also increase antibacterial activity, being effective in the eradication of MRSA biofilms [38,39].

Furthermore, the idea that the addition of a quinone moiety bearing a PEG group could target both bacterial reductive cytoplasm and the improvement of the water solubility of the phenazine, leading to the release of the active structure within the bacterial microenvironment, was investigated. Several carbonate-linked HP-quinone prodrugs (**46**–**49**, Figure 15) were developed and all exhibited a dramatic solubility enhancement when compared to the parent drug. Moreover, compound **46** was highlighted for its good stability and rapid activation into the active structure, presenting MIC values ranging from 0.78 to 1.17 µM against MRSA [38].

In the same year, nitroarene-based HP prodrugs bearing a sulfonate ester linker were synthesized. Through the action of intracellular nitroreductase, compound **50** (Figure 15) was able to release 70.1% of the active parent phenazine drug after 16 h, in vitro. Also, ideal antibacterial and good cytotoxicity profiles were found for this prodrug, with MIC values ranging from 3.13 to 6.25 µM against a panel of *S. aureus* strains, including MRSA [39].

**Scheme 4:**
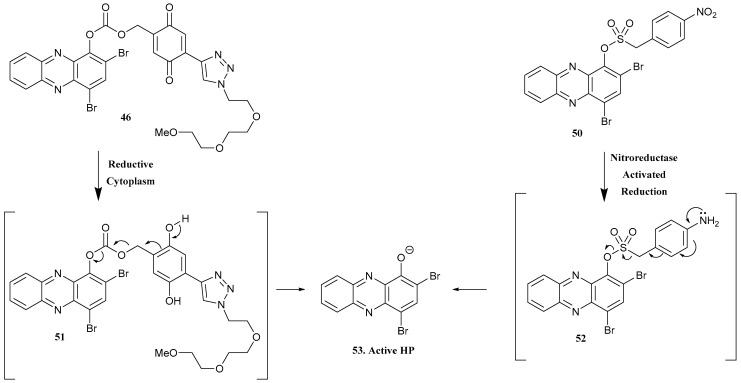
Mechanism of activation of HP-prodrugs **46** and **50** through intermediates **51** and **52**, respectively, into the active HP (53) [38,39].

The mechanism of activation of compounds **46** and **50** into the active HP-drug (**53**) is depicted in Scheme 4 [38,39].

**Figure 16:**
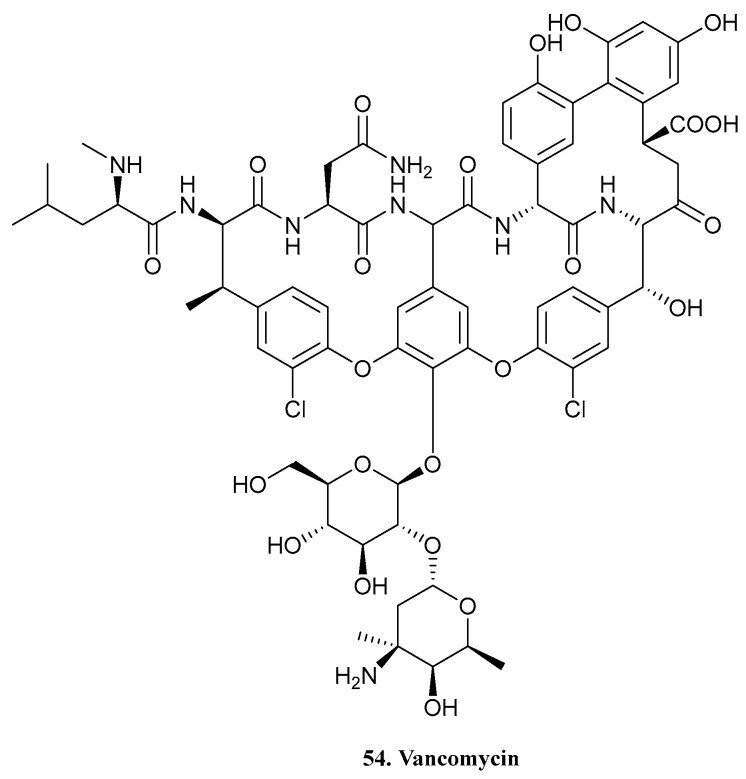
Structure of vancomycin (**54**) [41].

**Scheme 5:**
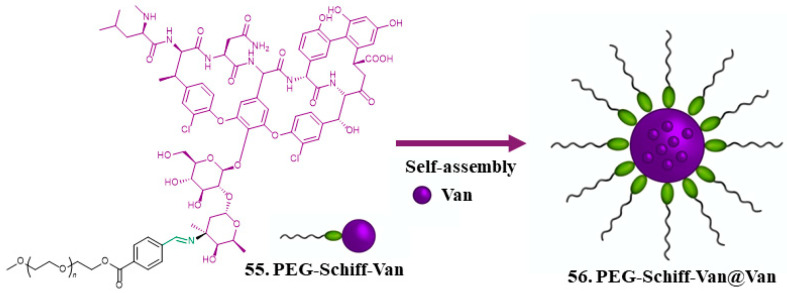
Structure and mechanism of self-assembly of the PEG-Schiff-Van conjugate (**55**) into PEG-Schif-Van@Van (**56**) nanoparticles. Vancomycin moiety is highlighted in purple and the pH-sensitive bond in green (inspired by the original illustration in [41]).

PEGylation of antibacterial agents has been used with a view to improving drugs’ half-lives in vivo, reducing toxicity, and increasing both solubility and stability [40]. In this context, Li et al. [14,41] developed a pH-responsive amphiphilic vancomycin conjugate prodrug system for the delivery of vancomycin (**54**, Van, Figure 16), a glycopeptide antibiotic. This system was composed of nanoparticles bearing Van derivatized with a PEG moiety and an acid liable Schiff base (PEG-Schiff-Van, **55**, Scheme 5) self-assembled into PEG-Schiff-Van@Van (**56**, Scheme 5). Inhibition zone measurements were performed at pH 7.4 and pH 6.6 for both the conjugate and bare Van after 6 and 24 h. At pH 7.4, a small zone of inhibition was observed within the initial 6 h. After 24 h of incubation, the diameter increased from 7 to 11 mm. In the more acidic medium, a larger zone of inhibition was evident during the first 6 h, having expanded to 12.5 mm after 24 h. Thus, these results suggested that the pH-sensitive Schiff base conferred system stability under neutral media, while promoting its disassembly under acidic bacterial conditions, leading to the release of free Van. Moreover, to assess the impact of the conjugate nanoparticles, in vivo assays were performed in a rat skin wound infection model with *S. aureus*. After 24 h of infection, wounds were treated with phosphate-buffered saline, Van, PEG-Schiff-Van, and PEG-Schiff-Van@Van. After 7 days, the wound treated with PEG-Schiff-Van@Van showed a significant reduction in size compared to the others, and by day 14 it had almost disappeared, while others were not completely healed.

**Figure 17:**
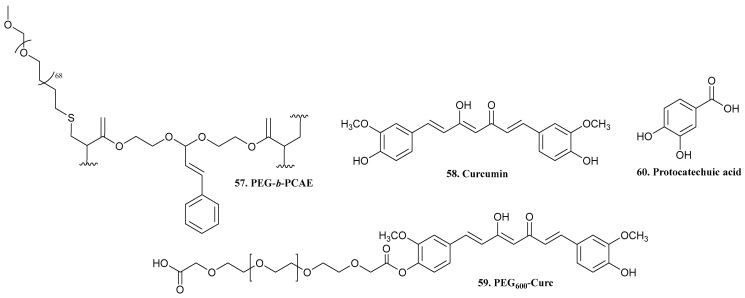
Structure of PEG-*b*-PCAE (**57**)**,** curcumin (**58**), PEG_600_-Curc complex (**59**), and protocatechuic acid (**60**) [42,43,44].

The use of natural antibacterial agents, such as cinnamaldehyde (CA) and curcumin (Curc), instead of conventional antibiotics has been studied [42,43]. CA has already shown potential activity against different GNB and GPB strains; however, its poor stability and lack of specificity towards infection sites have limited its therapeutical applications. Dai et al. [42] developed an amphiphilic prodrug system of PEG-*b*-poly[(3-phenylprop-2-ene-1,1-diyl)bis(oxy)bis(enthane-2,1-poly[(3-phenylprop-2-ene-1,1-diyl)bis(oxy)bis(enthane-2,1-diyl)diacrylate] (PEG-*b*-PCAE, **57**, Figure 17), which could self-assemble to form micelles to eradicate intracellular bacterial infections. Three different polymer micelles [PEG-*b*-PCAE1 (Mw = 7721 g/mol), PEG-*b*-PCAE2 (Mw = 10,949 g/mol), and PEG-*b*-PCAE3 (Mw = 14,829 g/mol)] were developed and tested. These pH-responsive micelles, upon macrophage-mediated delivery into the cell and acidic phagolysosome exposure, could release CA and damage the cell membrane, leading to bacterial death. Among these, PEG-*b*-PCAE2 exhibited the most promising antibacterial activity, demonstrating significant growth inhibition of *S. aureus* when its concentration reached 62.5 µg/mL, while showing negligible cytotoxicity. Moreover, it was also shown that in an *S. aureus* model in vivo, this nanocomplex was able to accelerate the wound healing process.

Curcumin (**58**, Figure 17) also displays several therapeutic properties, being recognized by its potent antibacterial activity against *S. aureus*, with a MIC value of 15 µg/mL. However, its poor bioavailability limits its applications [43]. In this context, Yakub et al. [43] introduced the development of a water-soluble and pH-stable conjugate prodrug, PEG_600_-Curc (**59**, Figure 17), which has antibacterial activity against GPB, including *S. aureus*. This conjugate exhibited a MIC value of 74 µg/mL, lower than that required to free Curc, which was attributed to the covalent bonding of Curc functional groups in the conjugate.

Furthermore, also in the context of natural product-derived prodrugs, AbouAitha et al. [44] combined protocatechuic acid (**60**, Figure 17), a natural antibacterial agent, with zinc oxide (ZnO) nanoparticles to create prodrug nanoformulations with antibacterial effects, including an effective inhibitor of *S. aureus* growth.

**Figure 18:**
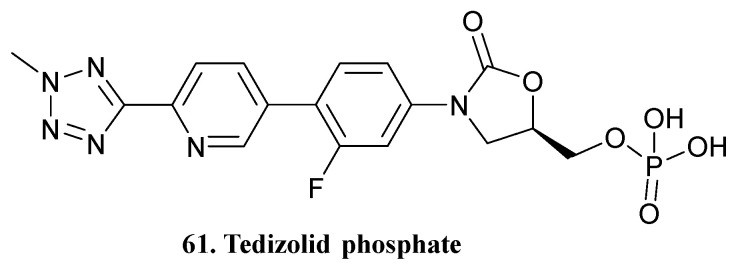
Structure of tedizolid phosphate (**61**) [45].

Tedizolid phosphate (TZP, **61**, Figure 18), belonging to the 1,3-oxazolidin-2-one class of antibiotics, is a tedizolid prodrug that may be used in the treatment of several MDR-bacterial infections, including ocular infections caused by MRSA. However, its poor ocular bioavailability limits its application. Thus, Alshmemry et al. [45] proposed the promising development of positively charged TZP nanocrystals for ocular delivery of the prodrug, resulting in better performance against *B. subtilis*, *S. pneumonia*, *S. aureus* and MRSA when compared to pure TZP.

**Figure 19:**
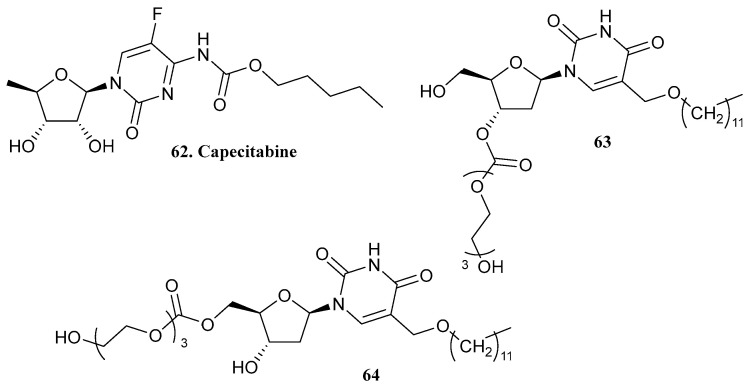
Structure of capecitabine (**62**) [46] and nucleoside-based prodrugs **63** and **64 [47]**.

In terms of repurposing commercially available drugs into antibacterial agents, capecitabine (**62**, Figure 19), an orally available anticancer fluorouracil prodrug with known activity against Gram-positive pathogens, was investigated by McLeod et al. [14,46]. The group tested this compound in a murine model infected with *S. aureus*, and the results showed that it was able to increase animal survival while reducing infected tissue colonization.

Nucleoside analogues, widely known for their antiviral and anticancer activity, may also become promising therapeutic agents against bacterial infections. In this context, in 2022, a small library of novel 3′-/5′-tri- or tetraethylene glycol prodrugs of 5-alkoxymethyl-2′-deoxyuridine was prepared. Synthesized compounds exhibited activity against various *M. smegmatis* and *S. aureus* bacterial strains, with compounds **63** (MIC = 41.5 µM for *M. smegmatis*; MIC = 83 µM for *S. aureus*, Figure 19) and **64** (MIC = 21 µM for *M. smegmatis*; MIC = 42 µM for *S. aureus*, Figure 19) showing the most potent antibacterial activity, alongside low cytotoxicity [14,47].

### 2.4. Prodrugs against Gram-Negative and Gram-Positive Bacteria

**Figure 20:**
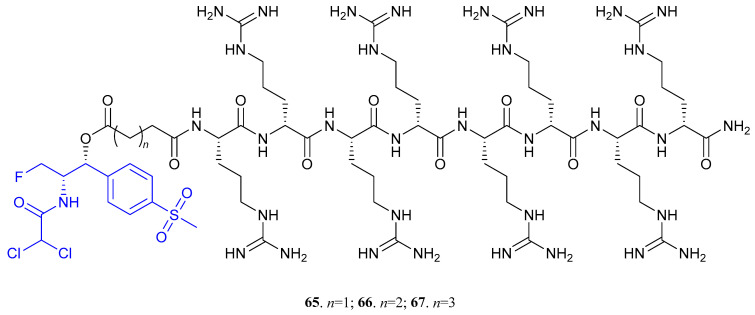
Structure of florfenicol-polyarginine conjugates **65**–**67**. Florfenicol moiety is highlighted in blue [48].

Florfenicol, belonging to the phenicol family of antibiotics, displays a broad antibacterial spectrum and good efficiency; however, its application is limited by its poor water-solubility [4,48]. Li et al. [48] developed a new set of florfenicol–polyarginine conjugates with selective toxicity towards bacterial cells when compared to mammalian cells, showing antibacterial activity in serum and plasma. Moreover, these conjugates, especially **65**–**67** (Figure 20), demonstrated excellent activity against MRSA and florfenicol-resistant *E. coli*, with MIC values of 12.5 µM against both strains, being able to decrease bacterial growth through membrane depolarization and disruption.

**Scheme 6:**
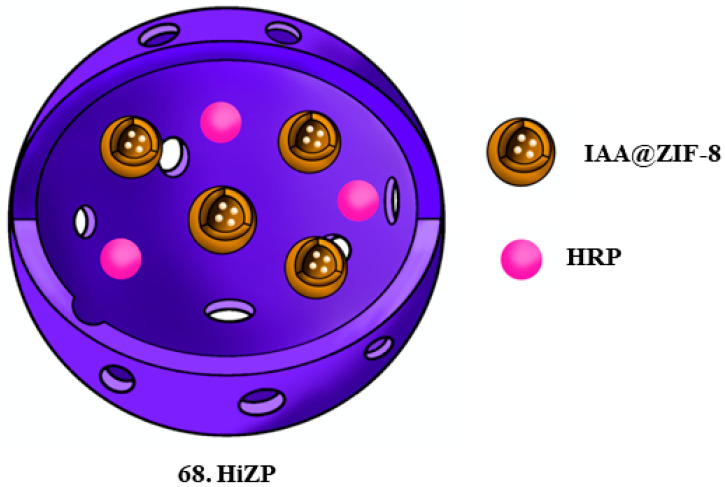
Illustration of the structure of HiZP (**68**) (inspired by the original illustration in [49]).

Weng et al. [49] developed an adenosine triphosphate-activated horseradish peroxidase (HRP)/indole-3-acetic acid (IAA) prodrug system. This system, consisting of the encapsulation of HRP and zeolitic imidazolate framework-8 (ZIF-8) loaded with IAA (IAA@ZIF-8) in polyacrylamide (pAAm), was given the name HRP&IAA@ZIF-8@pAAm or HiZP (**68**, Scheme 6). The mechanism of action of this system relies on the secretion of ATP by bacteria, leading to the decomposition of ZIF-8 and subsequent release of IAA. HRP is then expected to catalyze IAA’s oxidation, generating reactive oxygen species (ROS) responsible for bacterial membrane disruption. The developed system demonstrated the ability to destroy the bacterial membranes of *E. coli*, leading to its death. Moreover, a high antibacterial activity was detected against ampicillin-resistant *S. aureus* and ampicillin-resistant *E. coli*, demonstrating HiZP’s broad-spectrum antibacterial activity. Also, this system’s applicability in wound disinfection with minimal side effects was demonstrated.

**Figure 21:**
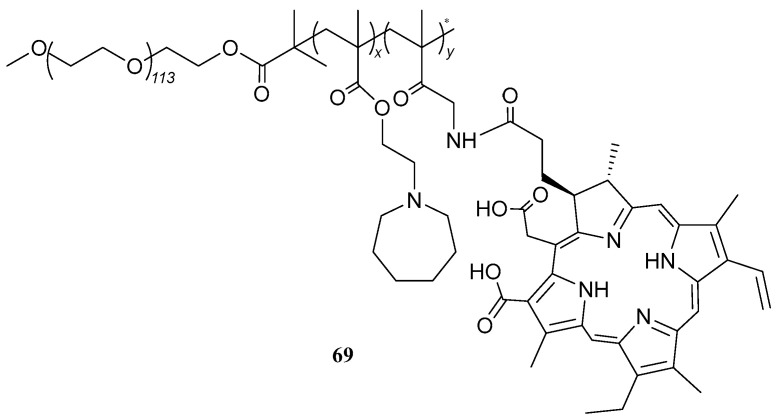
Structure of compound **69** synthesized by Weng et al. [50].

**Scheme 7:**
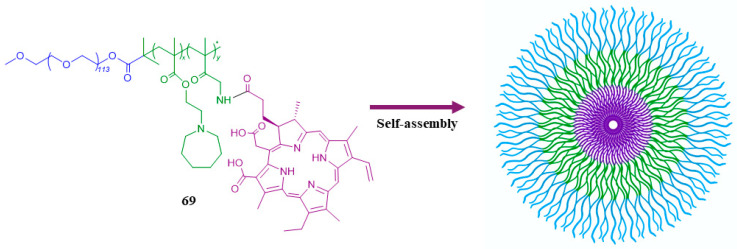
Illustration of the micelles formed by compound **69** (inspired by the original illustration in [50]).

In terms of nanotechnology, numerous studies on this topic were reported. In 2021, Wang et al. [50] synthesized and evaluated prodrug (**69**, Figure 21) forming micelles (Scheme 7). In MRSA and *E. coli* models, the incubation of varying micelle concentrations for 0.5 h led to a 99.84% reduction of MRSA at 8 µg/mL (pH 5.0), and a 99.46% reduction at 16 µg/mL (pH 7.4). For *E. coli*, achieving a 99.44% reduction at pH 5.0, a 32 µg/mL micelle concentration was required, while at pH 7.4 no significant antibacterial effect was detected. However, this system’s potential for broad-spectrum antibacterial action was highlighted. Furthermore, in in vivo experiments, the prodrug demonstrated sustained blood circulation and accumulation in the acidic bacterial microenvironment, making it highly efficient against MRSA infected mice.

The development of a bicomponent scaffold co-loaded with tetracycline hydrochloride (TH) and the prodrug indomethacin-PEG-indomethacin was reported by Cojocaru et al. [51] in 2023. This bicomponent system, bearing an electrospun outer membrane composed of chitosan/polyethylene oxide loaded with TH, and a 3D-printed gelatin composed of a methacryolyl/sodium alginate hydrogel loaded with the prodrug, exhibited outstanding activity against *S. aureus* and *E. coli* strains, demonstrating the promising use of electrospinning and 3D-printing technologies.

**Figure 22:**
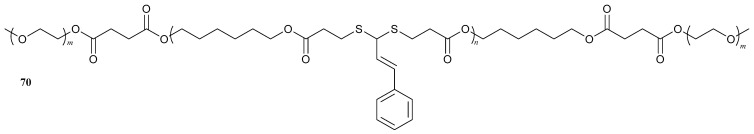
Structure of the cinnamaldehyde prodrug (**70**) [52].

In the same year, Lu’s group [52] reported the preparation of a novel prodrug system consisting of a PEGylated ROS-responsive polymeric prodrug of the natural antibacterial agent CA (**70**, Figure 22). This compound exhibited bactericidal effects against *E. coli* (MIC = 400 µg/mL) and *S. aureus* (MIC = 100 µg/mL) strains and, also, the degradation of its nanoparticles led to the “self-destruction” of the Gram-positive pathogens. Moreover, free CA exhibits MIC values of 160 µg/mL against *E. coli* and 80 µg/mL against *S. aureus* strains. However, the complete degradation of 400 µg/mL of prodrug nanoparticles would yield the release of 70.08 µg/mL of CA, a concentration significantly lower than its MIC value, suggesting the enhanced potency of the prodrug compared to free CA. Additionally, the prodrug exhibited good biocompatibility and reduced cytotoxicity when compared to the free drug.

**Figure 23:**
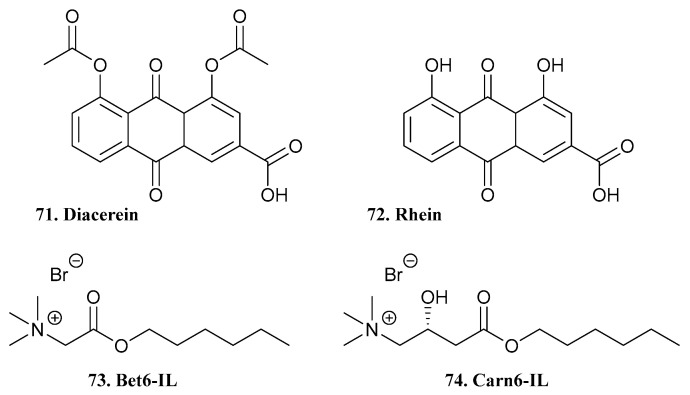
Structure of diacerein (**71**), rhein (**72**), Bet6-IL (**73**), and Carn9-IL (**74**) [53].

Diacerein (DIA, **71**, Figure 23) is an unstable anthraquinone prodrug with poor water solubility, hydrolyzed into active rhein (**72**, Figure 23), and commonly used in the treatment of osteoarthritis. In 2023, Grassiri et al. [53] proposed its repurposing as an antibacterial agent against ocular keratitis, often caused by *P. aeruginosa* and *S. aureus* bacterial strains. Hence, new eye-drops were formulated using betaine- and carnitine-based ionic liquids (IL) [Bet6-IL (**73**) and Carn6-IL (**74**), Figure 23] as excipients capable of self-assembling into nanoaggregates, promoting DIA’s solubilization and stability while enhancing its ocular residence time. Both DIA and rhein were tested for their efficacy against both bacterial strains, exhibiting MIC values of ≥50 µg/mL against clinical isolates of *S. aureus* W4 and *P. aeruginosa* ATCC 2783. Additionally, both Bet6 and Carn6 showed strong antibacterial effects against the tested strains.

In Table 1 the summary of Section 2 content is given, showing compounds’ antibacterial profiles and references.

## 3. Conclusions

Bacterial AMR represents an increasingly alarming threat that is expected to claim the lives of 10 million people annually by 2050 [9], requiring immediate attention. Thus, the development of new drugs is urgently required as bacteria continue to develop resistance mechanisms to existing treatments. In this context, exploring antibacterial prodrugs is emerging as a promising strategy to combat bacterial AMR. Prodrugs can overcome the sub-optimal pharmacokinetic, biophysical, and physiochemical properties that limit drug efficacy [11,12]. These compounds may address bacterial resistance by introducing new drug properties, new modes of action and new drug targets, in line with the WHO criteria defined for the development of new antibacterial agents [7]. However, developing prodrugs is challenging due to their unpredictable behavior compared to other drugs [11,12].

Herein, the most pertinent research conducted in the field of prodrug development from 2021 to 2023 is presented in four subsections: antituberculosis prodrugs, prodrugs against Gram-negative bacteria, prodrugs against Gram positive bacteria, and prodrugs targeting both Gram-negative and Gram-positive bacteria. Antibiotic structure modification, formulation of nanocomplexes, development of delivery systems, and repurposing of non-antibiotic drugs are amongst the methodologies described for prodrug production.

This review aims at offering a comprehensive understanding of the promise held by antibacterial prodrugs and prodrug systems as efficient tools to overcome bacterial resistance mechanisms and, as such, contributes to mitigating the escalating crisis posed by bacterial AMR.

## Figures and Tables

**Table 1:** Antibacterial compounds effective over Tuberculosis, GNB and GPB.

Compd. Nr.	Structure	Antibacterial Activity			Ref.
		Tuberculosis	GNB	GPB	
1. Isoniazid	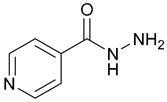	X	-	-	[16]
2. INH-HB	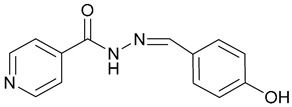	X	-	-	[18]
4. Ethionamide	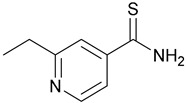	X	-	-	[19]
5.SMARt751	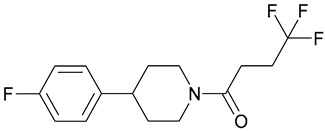				
6. SPR720	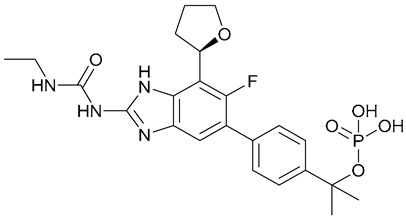	X	-	-	[20]
7	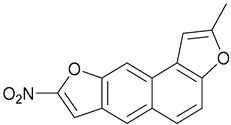	X	-	-	[21]
8	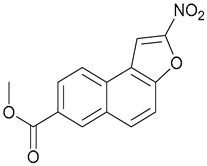				
9. Aminopyrro- lidine	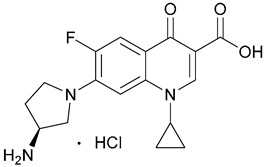	-	X	-	[22]
10. Aminomethylpyrro-lidine	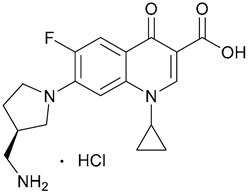				
11. Ciprofloxacin	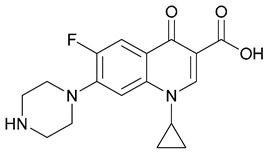				
12	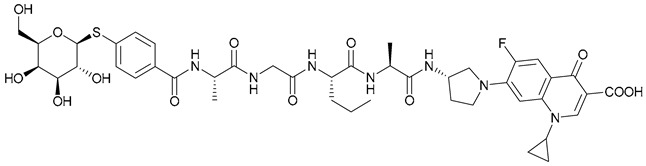	-	X	-	[22]
13	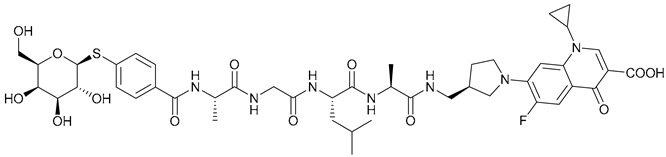				
14	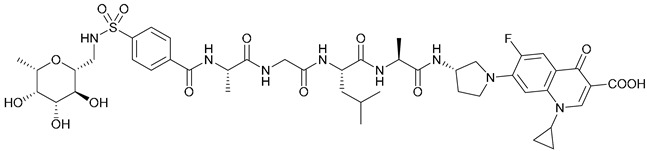				
15	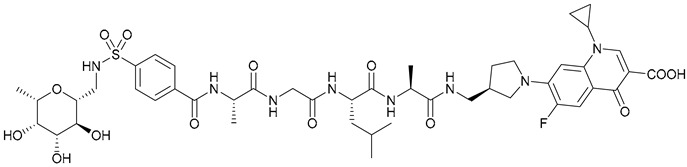				
16. Moxifloxacin	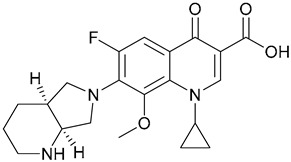	-	X	-	[23]
17. *N*-Moxi	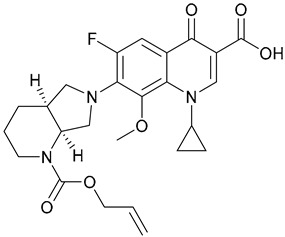				
18. *C*-Moxi	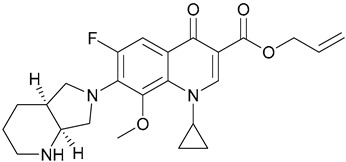				
22. TBP-PI-HBr	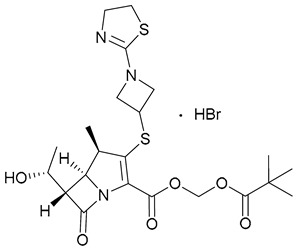	-	X	-	[24,25,26,27,28]
23. Ceftibuten	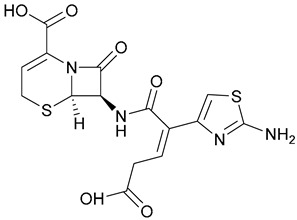	-	X	-	[29,30]
24. Avibactam	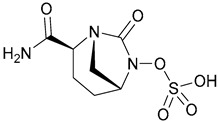				
25. Rifabutin	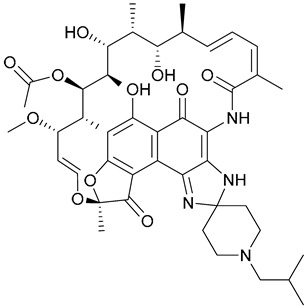	-	X	-	[31]
26	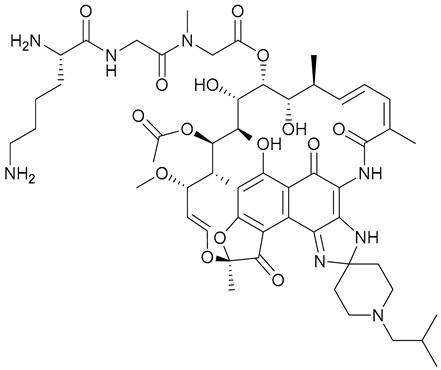				
27	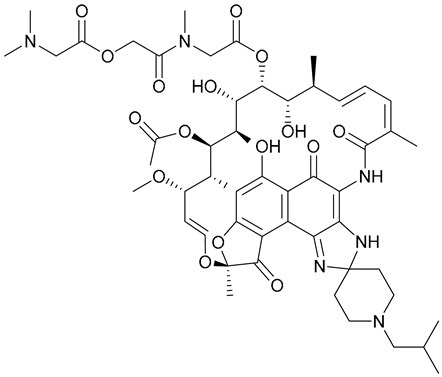				
28	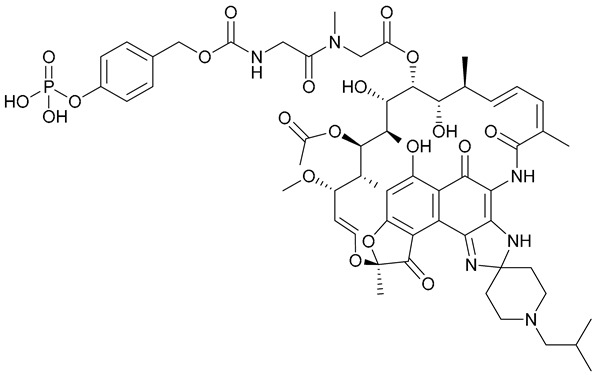				
29. Tobramycin	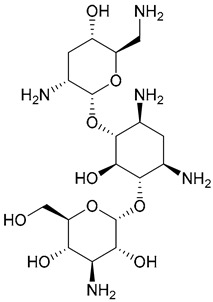	-	X	-	[32]
30. Azithromycin	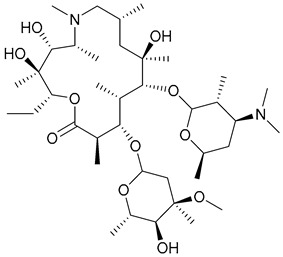				
33. Colistin methanesulfonate	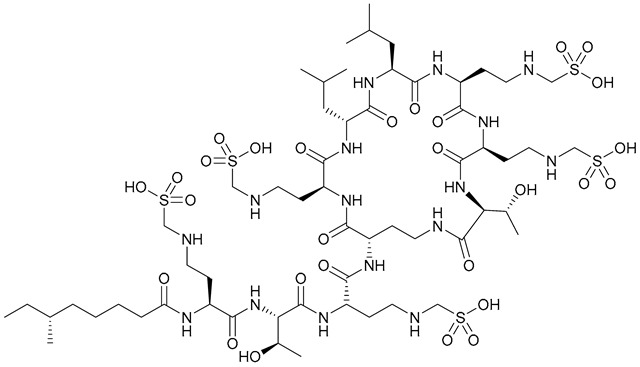	-	X	-	[34]
34. pEt_20	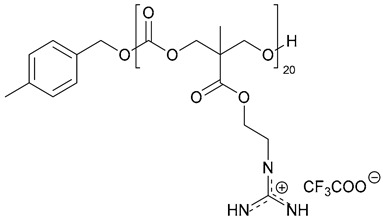				
38, 39. l-EP/d-EP	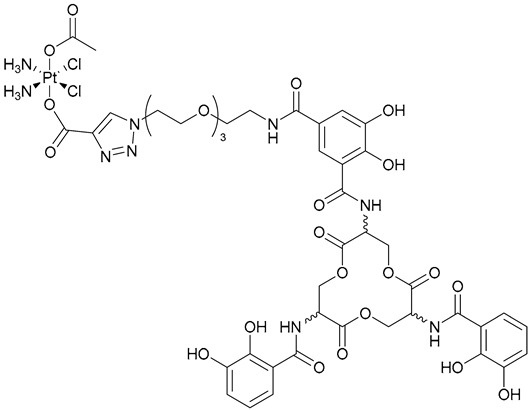	-	X	-	[35]
40. Zidovudine	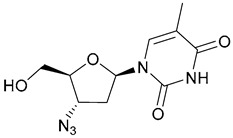	-	X	-	[36]
41	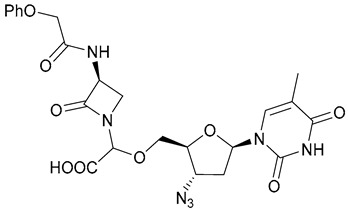				
42	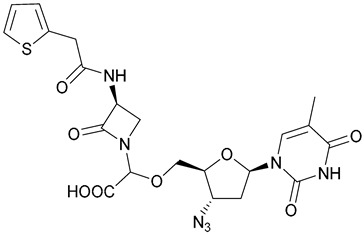				
43. Oxacillin	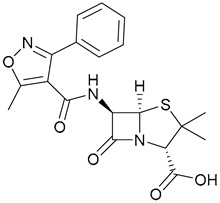	-	-	X	[37]
44. TXA709	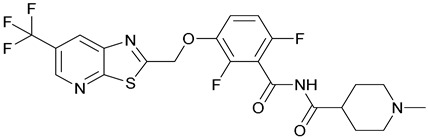				
45. Marine Phenazine	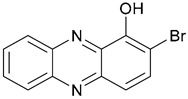	-	-	X	[38]
46	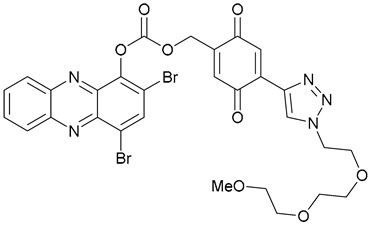				
47	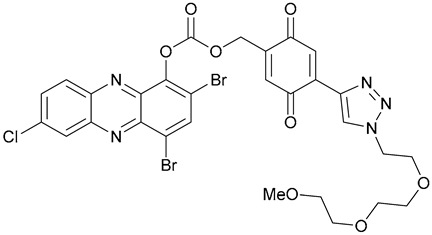				
48	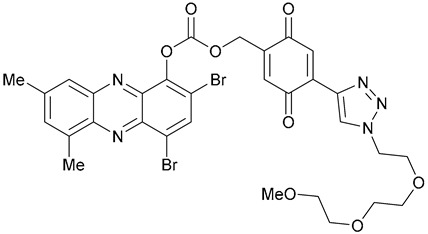				
49	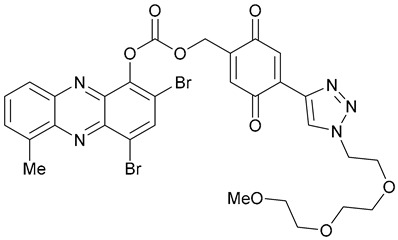				
50	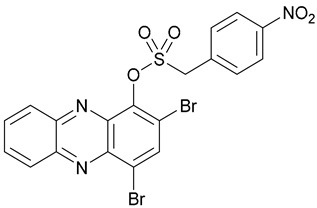	-	-	X	[39]
54. Vancomycin	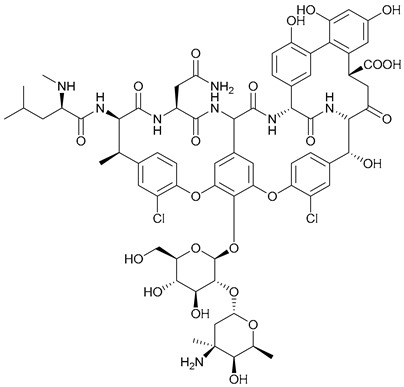	-	-	X	[41]
55. PEG-Schiff -Van	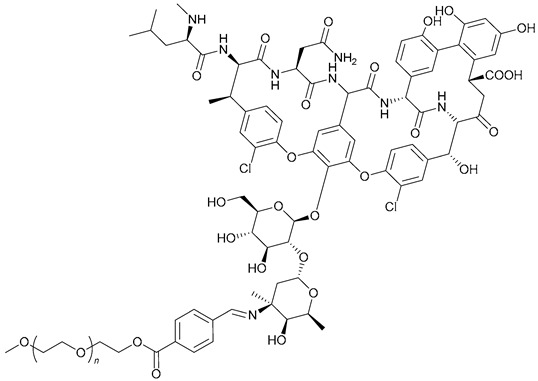				
57. PEG-*b*-PCAE	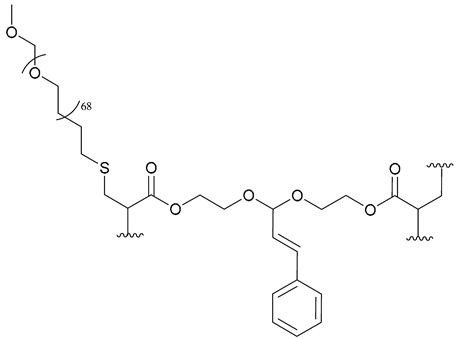	-	-	X	[42]
58. Curcumin	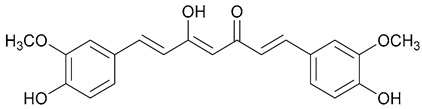	-	-	X	[43]
59. PEG_600_-Curc	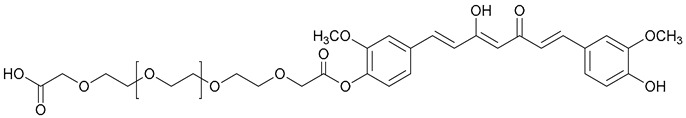				
60. Protocatechuic acid	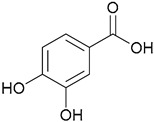	-	-	X	[44]
61. Tedizolid phosphate	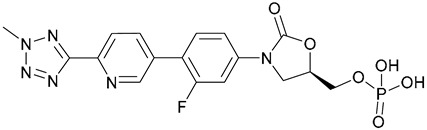	-	-	X	[45]
62. Capecitabine	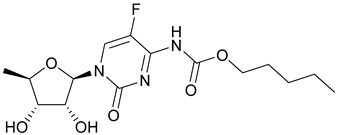	-	-	X	[46]
63	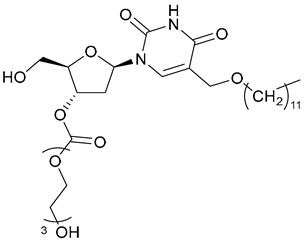	-	-	X	[47]
64	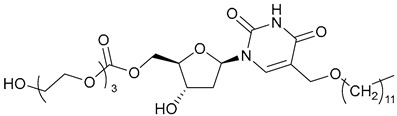				
65 (*n* = 1); 66 (*n* = 2); 67 (*n* = 3)	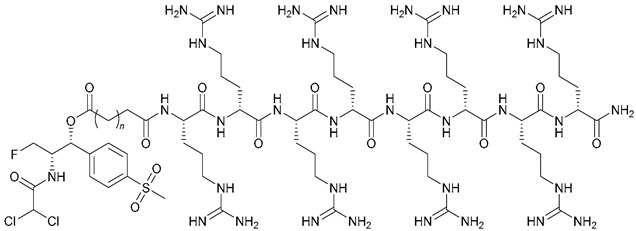	-	X	X	[48]
68. HiZP	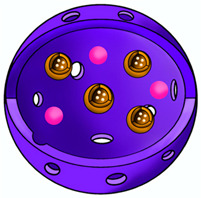	-	X	X	[49]
69	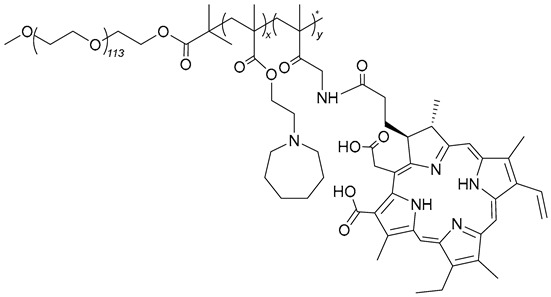	-	X	X	[50]
70	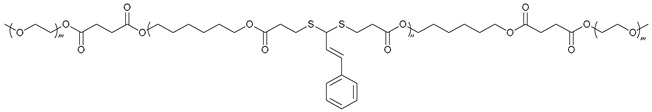	-	X	X	[52]
71. Diacerin	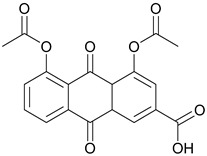	-	X	X	[53]
72. Rhein	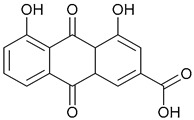				
73. Bet6-IL	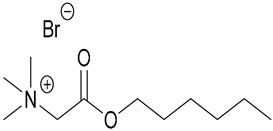				
74. Carn6-IL	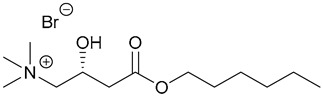				

## Data Availability

Not applicable.
